# *DOK2* Inhibits *EGFR*-Mutated Lung Adenocarcinoma

**DOI:** 10.1371/journal.pone.0079526

**Published:** 2013-11-08

**Authors:** Alice H. Berger, Ming Chen, Alessandro Morotti, Justyna A. Janas, Masaru Niki, Roderick T. Bronson, Barry S. Taylor, Marc Ladanyi, Linda Van Aelst, Katerina Politi, Harold E. Varmus, Pier Paolo Pandolfi

**Affiliations:** 1 Cancer Genetics Program, Beth Israel Deaconess Cancer Center, Departments of Medicine and Pathology, Beth Israel Deaconess Medical Center, Harvard Medical School, Boston, Massachusetts, United States of America; 2 Cancer Biology and Genetics Program, Sloan-Kettering Institute, Memorial Sloan-Kettering Cancer Center, New York, New York, United States of America; 3 Cold Spring Harbor Laboratory, Cold Spring Harbor, New York, United States of America; 4 Department of Internal Medicine, University of Iowa, Iowa City, Iowa, United States of America; 5 Rodent Histopathology Core, Dana-Farber/Harvard Cancer Center, Boston, Massachusetts, United States of America; 6 Computational Biology Center, Memorial Sloan-Kettering Cancer Center, New York, New York, United States of America; 7 Department of Pathology, Memorial Sloan-Kettering Cancer Center, New York, New York, United States of America; 8 Human Oncology and Pathogenesis Program, Memorial Sloan-Kettering Cancer Center, New York, New York, United States of America; Consiglio Nazionale delle Ricerche (CNR), Italy

## Abstract

Somatic mutations in the *EGFR* proto-oncogene occur in ~15% of human lung adenocarcinomas and the importance of *EGFR* mutations for the initiation and maintenance of lung cancer is well established from mouse models and cancer therapy trials in human lung cancer patients. Recently, we identified *DOK2* as a lung adenocarcinoma tumor suppressor gene. Here we show that genomic loss of *DOK2* is associated with *EGFR* mutations in human lung adenocarcinoma, and we hypothesized that loss of *DOK2* might therefore cooperate with *EGFR* mutations to promote lung tumorigenesis. We tested this hypothesis using genetically engineered mouse models and find that loss of *Dok2* in the mouse accelerates lung tumorigenesis initiated by oncogenic *EGFR*, but not that initiated by mutated *Kras*. Moreover, we find that DOK2 participates in a negative feedback loop that opposes mutated *EGFR*; *EGFR* mutation leads to recruitment of DOK2 to EGFR and DOK2-mediated inhibition of downstream activation of RAS. These data identify *DOK2* as a tumor suppressor in *EGFR*-mutant lung adenocarcinoma.

## Introduction

*EGFR* and *KRAS* mutations are the two most frequent oncogenic events in human lung adenocarcinoma, occurring in approximately 15% and 30% of U.S. lung adenocarcinoma cases, respectively [1]. Somatic mutation of *EGFR* defines a specific subclass of lung adenocarcinomas with sensitivity to treatment with the EGFR inhibitors gefitinib or erlotinib [2-4]. The two major classes of *EGFR* mutations are an L858R point mutation and small, in-frame deletions in exon 19; both types of mutation enhance the activity and oncogenicity of EGFR compared to the wild-type protein [5]. Tumors harboring *KRAS* mutations are found more frequently in smokers and predict primary resistance to targeted EGFR inhibitors, whereas mutations in *EGFR* are more frequent in women, never-smokers, and East Asian populations, and predict sensitivity to *EGFR* kinase inhibitors [3,6-8]. 

 The “downstream of tyrosine kinase” (DOK) proteins are a family of adaptor proteins that modulate tyrosine kinase signaling. Similar to the insulin receptor substrate (IRS) proteins, the seven DOK family members contain an N-terminal pleckstrin homology (PH) domain, a phospho-tyrosine binding (PTB) domain, and a C-terminus containing numerous tyrosine residues and proline-rich motifs. Upon growth factor stimulation, DOK proteins are localized to membrane signaling complexes via interactions involving the DOK PH and PTB domains, where they recruit additional proteins through interactions of the phospho-tyrosine residues and PXXP motifs on the DOK C-terminus with SH2 and SH3 domains, respectively [9-11]. DOK1, DOK2, and DOK3 regulate numerous downstream targets of RTKs including AKT, SRC, and RAS by functioning as inducible adaptors that recruit negative signaling regulators into the signaling complex [9,10,12-15]. For example, DOK1 and DOK2 function upstream of RAS and inhibit RAS activity by enhancing the recruitment of the RAS GTPase activating protein RASA1/RASGAP to RAS [10,16]. 

In addition to other RTK pathways, DOK proteins are able to regulate signaling downstream of EGFR. Both DOK1 and DOK2 are phosphorylated after EGF stimulation and can bind directly to phosphotyrosines on EGFR [12,17,18]. Moreover, DOK2 has been shown to suppress SRC, AKT, and ERK phosphorylation after EGF stimulation [12]. Given these data, as well as our recent identification of *DOK2* as a human lung tumor suppressor gene [19], we sought to test whether perturbation of *DOK2* in human and mouse lung cell lines or transgenic mice would alter *EGFR*- or *KRAS-*mutant lung tumorigenesis.

## Results

### Association between genomic loss of DOK2 and mutation of EGFR in human lung adenocarcinoma

**Figure 1:**
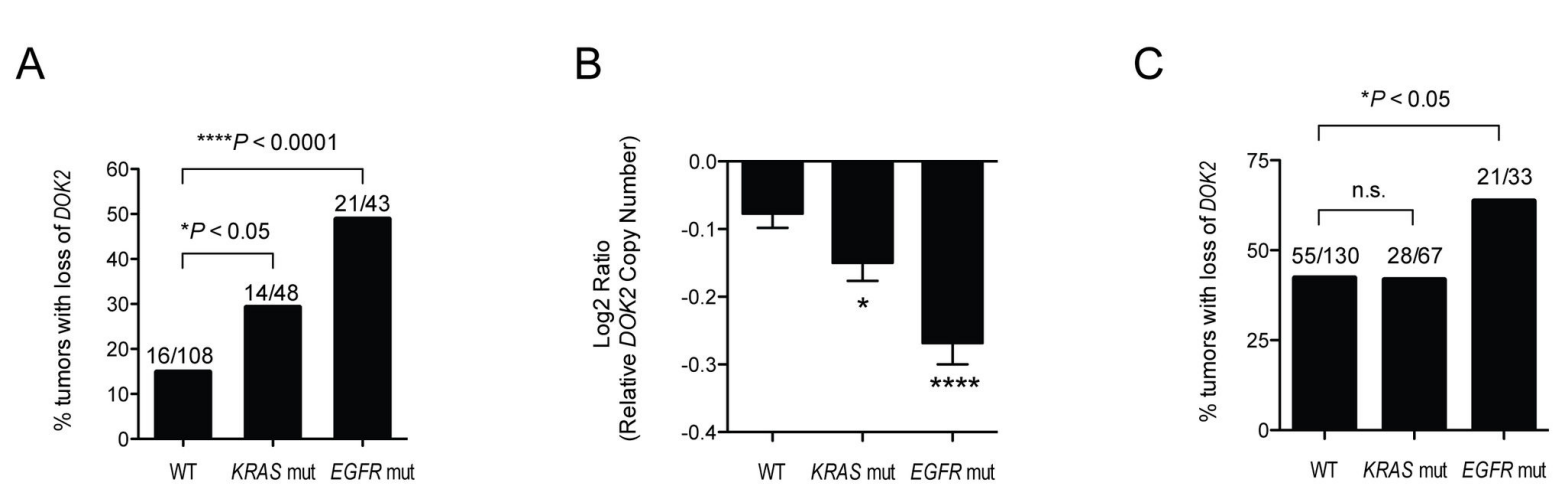
Loss of *DOK2* in human lung adenocarcinoma is associated with *EGFR* mutation. (**A**) Association between *EGFR* or *KRAS* mutation status and genomic loss of the *DOK2* locus from aCGH analysis of 199 primary human lung adenocarcinomas [20]. ****, *P* < 0.001. *, P < 0.05 by Fisher’s exact test. aCGH analysis and mutation calling of tumors was determined as previously described [20]. (**B**) Quantitative representation of data shown in (A). Data shown is mean+SEM of log_2_ ratio data from array CGH data. ***, *P* < 0.001. *, *P* < 0.05 by two-tailed unpaired t-test. (**C**) Copy number and mutation associations in The Cancer Genome Atlas (TCGA) data from 230 lung adenocarcinomas. Copy number and mutation data were downloaded from TCGA (https://tcga-data.nci.nih.gov/).

We previously identified *Dok* family genes as murine lung tumor suppressors and *DOK2* as a candidate human lung tumor suppressor gene [19]. *DOK2* expression is downregulated in human lung adenocarcinoma due to heterozygous genomic loss encompassing the *DOK2* gene at the 8p21.3 locus [19]. To determine if *DOK2* genomic loss was a feature of a specific genomic class of lung adenocarcinoma, we analyzed the relationship of *DOK2* loss with mutation of *EGFR* or *KRAS* in 199 primary human lung adenocarcinomas [19,20]. Interestingly, loss of *DOK2* strongly correlated with *EGFR* mutation status; tumors with an *EGFR* mutation had a significantly elevated frequency of loss of *DOK2* (Figure 1A-B, *P* < 0.0001). Moreover, we observed the same association in recent data generated by The Cancer Genome Atlas (TCGA) from 230 lung adenocarcinomas (Figure 1C). There was a weak but significant association between loss of *DOK2* and *KRAS* mutation (Figure 1A-B, *P* < 0.05). However, only the association with *EGFR* mutation was replicated in the TCGA data, suggesting loss of *DOK2* is associated with *EGFR* mutation but not *KRAS* mutation in human lung adenocarcinoma.

The observed genetic association is consistent with selection for *DOK2* loss in *EGFR*-mutant tumors. However, many other genes reside in the region of 8p that encompasses the *DOK2* gene, raising the possibility that selection for loss of other genes could be responsible for the observed association. For example, *DOK2* lies telomeric to *DUSP4*, a MAP-kinase phosphatase which negatively regulates MAPK signaling downstream of RAS and has been identified as a candidate tumor suppressor gene [20]. Thus we performed additional *in vitro* and *in vivo* experiments to determine whether *DOK2* could inhibit *EGFR*- or *KRAS*-mutant lung tumorigenesis. 

### DOK2 suppresses EGF-induced RAS and ERK activity and constitutively interacts with EGFR^L858R^

**Figure 2:**
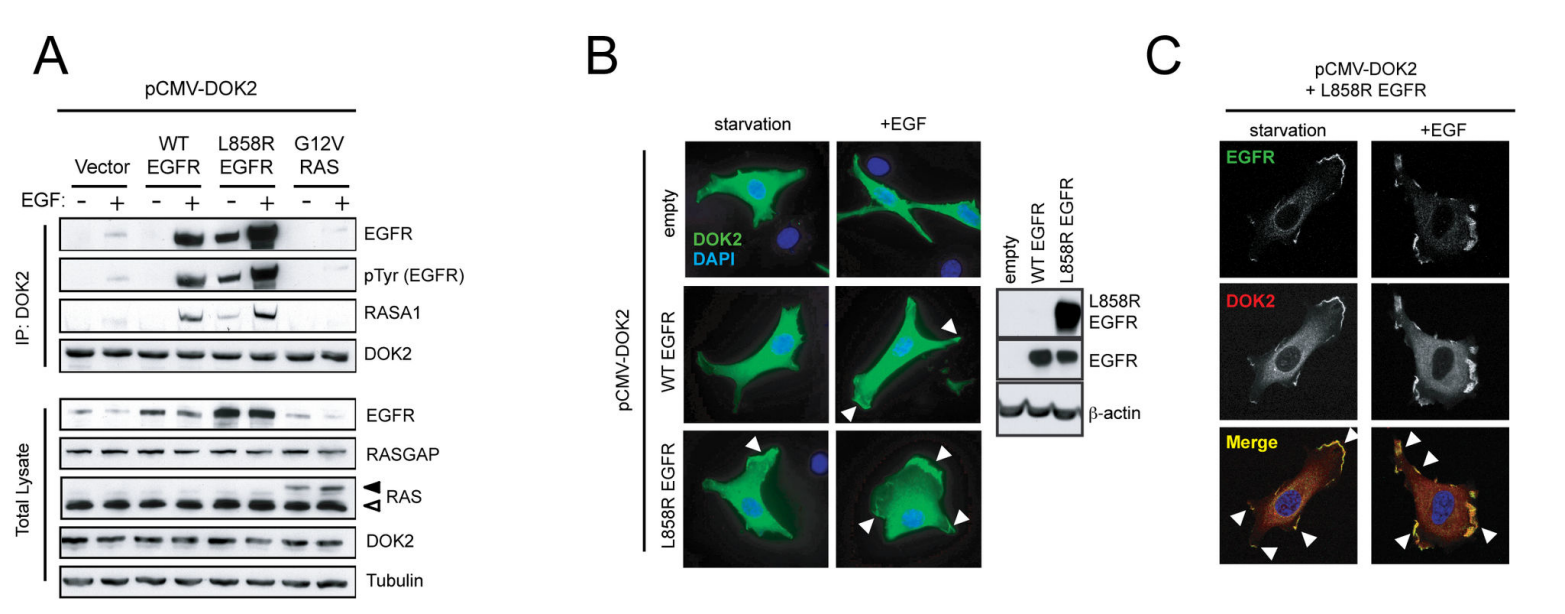
EGFR activity regulates binding of DOK2 to EGFR and DOK2 localization. (**A**) Co-immunoprecipitation of DOK2, EGFR, and RASA1. HEK293 cells were co-transfected with pCMV-DOK2 (all conditions) and either pcDNA3.1 empty vector or pcDNA3.1-EGFR^WT^, pcDNA3.1-EGFR^L858R^, or pcDNA3.1-HA-HRAS^G12V^. Cells were serum starved overnight in medium containing 0.1% FBS before stimulation with 50 ng/ml EGF for 2-5 minutes. DOK2 and associated proteins were immunoprecipitated from extracts of stimulated and unstimulated cells with an anti-DOK2 antibody and then analyzed by Western blotting. Upper panels show the proteins in the immunoprecipitation fraction; lower panels show proteins from the total lysate. A black arrowhead indicates HA-HRAS^G12V^ whereas a white arrowhead indicates endogenous RAS. Data shown is representative of results from at least three independent experiments. (**B**) Immunofluorescence of NIH3T3 cells stably expressing WT or EGFR^L858R^. Cells were transfected with pCMV-DOK2, incubated overnight, serum starved, and then either fixed or stimulated with EGF before fixation. The left panels show DOK2 (green) and DAPI (blue). White arrowheads indicate membrane-associated DOK2 staining. A Western blot indicates total levels of EGFR in these cells (right panel). Data shown is representative from at least three independent experiments. (**C**) Confocal microscopy analysis of colocalization of DOK2 and EGFR. 3T3-EGFR^L858R^ cells were transfected with FLAG-DOK2 and probed with anti-FLAG (red) or anti-EGFR (green) antibodies. Arrowheads indicate colocalization (yellow) in the panel showing the merged signals. Data shown is representative from at least three independent experiments.

First, using a RAS-binding domain (RBD) pull-down assay and Western blotting, we confirmed that overexpression of DOK2 suppresses EGF-induced RAS and ERK activity in HEK293T cells compared to cells transfected with empty vector (Figure S1). Next, we examined the physical interaction between DOK2 and EGFR. We overexpressed DOK2 alone or in combination with human wild-type EGFR, EGFR^L858R^, or RAS^G12V^. Under serum starvation conditions, no binding of DOK2 to wild-type EGFR was observed (Figure 2A). EGF stimulation induced interactions of DOK2 and wild-type EGFR and RASA1 (Figure 2A). In contrast, DOK2 physically interacted with EGFR^L858R^ and RASA1 not only after EGF stimulation but also during serum-starvation (Figure 2A). Therefore activation of EGFR, either ligand-induced or as a consequence of oncogenic mutation appears to induce DOK2 recruitment to the EGF receptor complex and DOK2-mediated recruitment of RASA1. 

### EGFR activation regulates localization of DOK2

Next, we used immunofluorescence to examine localization of DOK2 with or without EGF stimulation in NIH3T3 fibroblasts stably overexpressing wild-type EGFR or EGFR^L858R^ and transfected with a DOK2 expression construct. In cells lacking expression of EGFR, DOK2 was diffusely distributed in the cytoplasm during serum starvation and after EGF stimulation (Figure 2B). In cells expressing wild-type EGFR, however, DOK2 localization changed from diffusely cytoplasmic to markedly localized at the plasma membrane after EGF stimulation, consistent with recruitment of DOK2 to the receptor complex after autophosphorylation of EGFR (Figure 2B). In cells expressing EGFR^L858R^, DOK2 was present at the membrane not only after EGF stimulation but also during serum-starvation (Figure 2B), indicating that activation of EGFR alone is sufficient to induce DOK2 localization at the plasma membrane. Confocal microscopy confirmed that DOK2 and EGFR co-localized in these membrane regions (Figure 2C). These results thus corroborate our biochemical observation in HEK293 cells and demonstrate that activation of EGFR induces relocation of DOK2 to the plasma membrane, where DOK2 binds to a protein complex containing EGFR (Figure 2A, C).

### DOK2 inhibits expansion of EGFR-mutant, but not KRAS-mutant, lung adenocarcinoma cells

**Figure 3:**
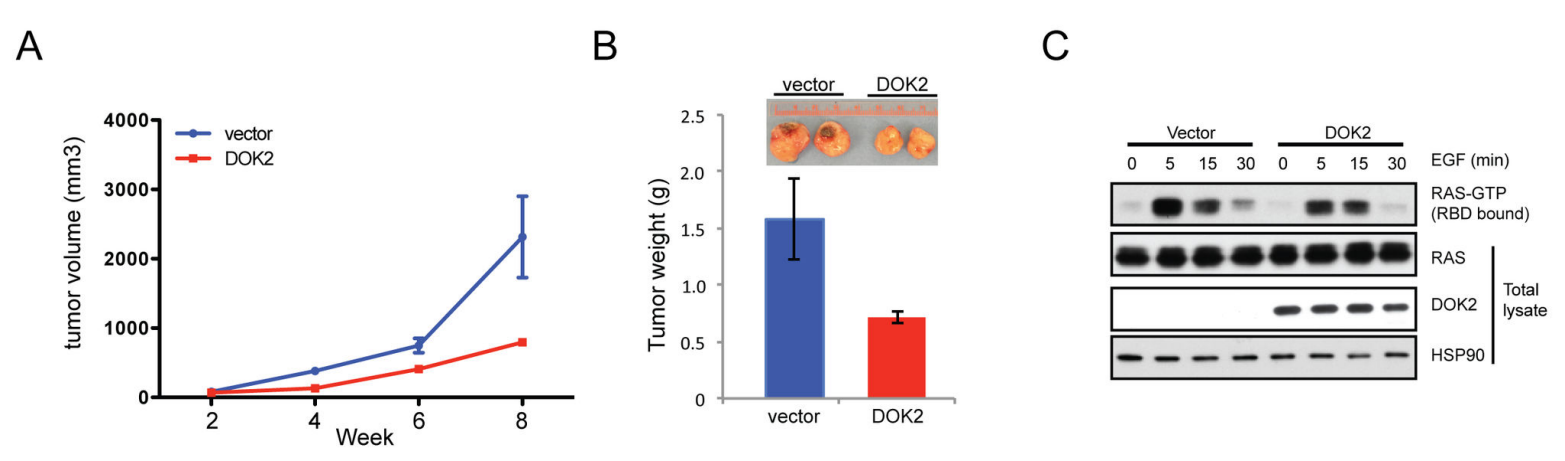
*DOK2* inhibits tumor formation of *EGFR*-mutant lung adenocarcinoma cells. (**A**) Tumor volume of NCI-H1975 cells after xenografting into nude mice. NCI-H1975 cells were transduced with retrovirus to generate stable cell lines with or without expression of DOK2 and each line was then injected subcutaneously into the flanks of nude mice. Data shown are mean +/- SD of three replicates. (**B**) Weight of tumors formed by NCI-H1975 cells with or without expression of DOK2. Pictures (inset) were taken at 8 weeks post-injection. (**C**) RAS activity in NCI-H1975 cells with or without DOK2 expression. The cells were serum starved and then stimulated with 100 ng/ml EGF for the indicated time, then lysed. GTP-bound RAS was isolated from lysates via a RAF-binding domain (RBD) pulldown, and the pulldown fraction (top panel) or total lysate (bottom panels) were analyzed by Western blotting using anti-RAS, DOK2, or HSP90 (loading control) antibodies.

**Figure 4:**
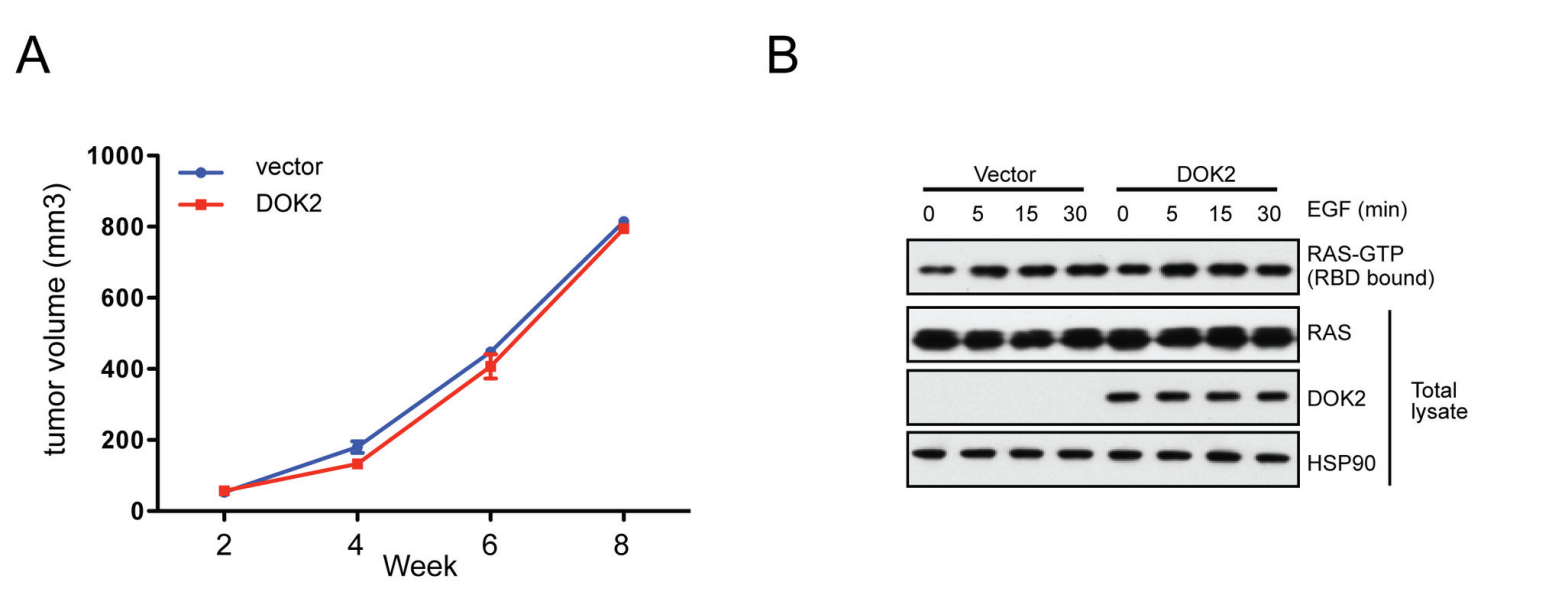
*DOK2* fails to inhibit tumor formation of *KRAS*-mutant lung adenocarcinoma cells. (**A**) Tumor volume of A549 cells after xenografting into nude mice. A549 cells were transduced with retrovirus to generate stable cell lines with or without expression of DOK2 and then each line was injected subcutaneously into the flanks of nude mice. Data shown are mean +/- SD of three replicates. (**B**) RAS activity in A549 cells with or without DOK2 expression. The cells were serum starved and then stimulated with 100 ng/ml EGF for the indicated time, then lysed. GTP-bound RAS was isolated from lysates via a RAF-binding domain (RBD) pulldown, and the pulldown fraction (top panel) and total lysate (bottom panels) were analyzed by Western blotting using anti-RAS, DOK2, or HSP90 (loading control) antibodies.

Next we sought to determine if *DOK2* could inhibit the growth of *EGFR*-mutant human lung adenocarcinoma cells. To this end, we ectopically expressed *DOK2* in NCI-H1975 cells, which contain both the L858R point mutation of *EGFR* and also a T790M mutation, the “gatekeeper” mutation in *EGFR* that confers resistance to first-generation EGFR inhibitors [8,21]. We then analyzed the effect of *DOK2* expression on tumor formation after subcutaneous xenograft into immunocompromised mice (Figure 3A-B). DOK2 expression inhibited tumor formation (Figure 3A-B) and partially suppressed EGF-induced RAS activation in NCI-H1975 cells (Figure 3C). In contrast, ectopic expression of DOK2 in *KRAS*-mutant A549 lung adenocarcinoma cells did not suppress tumor growth (Figure 4A) or activation of RAS (Figure 4B). These data are consistent with the model that DOK2 functions downstream of EGFR but upstream of RAS to suppress signal transduction and oncogenesis.

### Loss of Dok2 cooperates with EGFR mutation, but not Kras mutation, to promote lung tumorigenesis *in vivo*

To determine if loss of *DOK2* cooperates with *EGFR* or *KRAS* mutation *in vivo*, we crossed *Dok2* knockout (KO) mice [14] to lung-specific, doxycycline-inducible bitransgenic EGFR (*C/EGFR* ^*DEL*^) and Kras (*C/Kras* ^*G12D*^) mouse models [22,23]. Cohorts of mice in either a *Dok2* wild-type or *Dok2* KO background were placed on a diet containing doxycycline at weaning age and tumor formation was compared in the presence and absence of *Dok2*. Non- or mono-transgenic animals that did not express the oncogenic transgenes were used as controls. 

**Figure 5:**
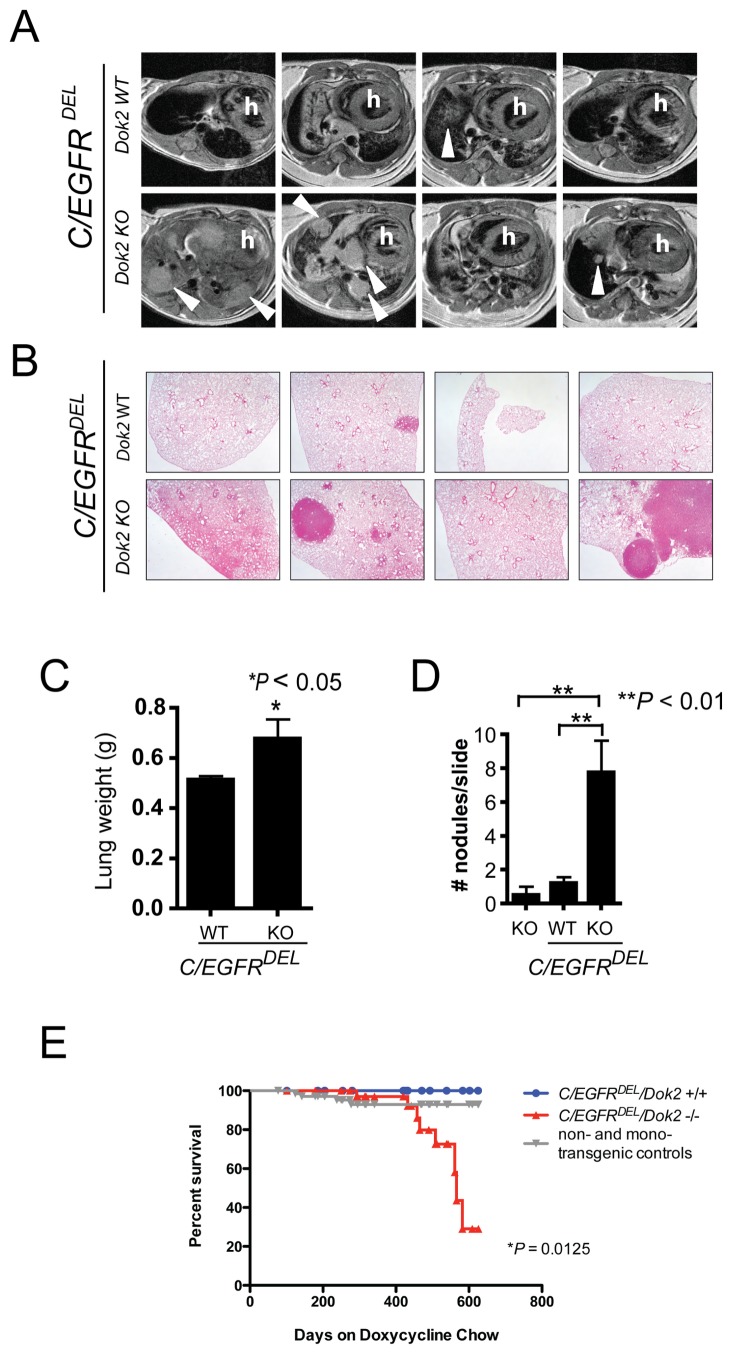
*Dok2* suppresses lung tumorigenesis initiated by oncogenic *EGFR.* (**A**) MR images from the lungs of *C/EGFR^DEL^/Dok2* ^*+/+*^ and *C/EGFR^DEL^/Dok2* ^*-/-*^ mice after 12 months of doxycycline treatment. Images from four individual animals of each genotype are shown. Arrowheads indicate tumor nodules. h, heart. Signal not indicated by arrows or arrowheads is likely to be diffuse hyperplasia or bronchoalveolar carcinoma (BAC). (**B**) H&E staining of lungs from *C/EGFR^DEL^/Dok2* ^*+/+*^ and *C/EGFR^DEL^/Dok2* ^-/-^ mice after 12 months of doxycycline treatment. 20X total magnification. For each genotype, four lung lobes from a single representative mouse are shown. (**C**) Lung weight of lungs from *C/EGFR^DEL^/Dok2* ^*+/+*^ and *C/EGFR^DEL^/Dok2* ^*-/-*^ mice after 12 months of doxycycline treatment. Data shown is mean + SEM. *, *P* < 0.05 by two-tailed t-test. *C*/EGFR^DEL^/Dok2^+/+^, n = 8. *C/EGFRDEL/Dok2* ^*-/-*^, n = 5. (**D**) Tumors per slide per animal in *C/EGFR^DEL^/Dok2* ^*+/+*^, *C/EGFR^DEL^/Dok2* ^*-/-*^, and age-matched non-transgenic *Dok2* KO mice. Data shown is mean + SEM. **, *P* < 0.01 by two-tailed t-test. *C*/EGFR^DEL^/Dok2^+/+^, n = 5. *C/EGFRDEL/Dok2* ^*-/-*^, n = 4. Non-transgenic *Dok2* KO, n = 4. (**E**) Kaplan-Meier plot of survival data from bitransgenic *C*/EGFR^DEL^/Dok2^+/+^ (n = 29), *C*/EGFR^DEL^/Dok2^-/-^ (n = 39), and non- or mono-transgenic littermate controls of all *Dok2* genotypes (n = 71). Spontaneous deaths or sacrifices due to poor body condition were recorded as events. Planned sacrifices at other time points were censored.

We monitored tumor formation and progression in *Dok2* wild-type or *Dok2* KO (-/-) *C/EGFR* ^*DEL*^ bitransgenic mice using magnetic resonance (MR) imaging at intervals over the course of one year. After 12 months of doxycycline induction, tumors were clearly visible in the *C/EGFR^DEL^/Dok2* ^*-/-*^ cohort with the MR images from *C/EGFR^DEL^/Dok2* ^*-/-*^ mice showing markedly more and larger tumors than seen in the lungs from *C*/EGFR^DEL^/Dok2^+/+^ mice (Figure 5A, n = 4 *Dok2* ^*-/-*^ and n = 5 *Dok2* ^*+/+*^). Upon pathological examination, lungs from *C/EGFR^DEL^/Dok2* ^-/-^ animals had a significantly greater number of tumors compared to lungs from *C/EGFR^DEL^/Dok2* ^*+/+*^ mice (Figure 5B,D). In agreement with these observations, the weight of lungs from *C/EGFR^DEL^/Dok2* ^*-/-*^ mice was significantly greater than that of lungs from the *C/EGFR^DEL^/Dok2* ^*+/+*^ controls (Figure 5C). *Dok2* KO mice without *EGFR* expression displayed many fewer tumor nodules at this age than *C/EGFR* ^*DEL*^ mice, indicating that *Dok2* loss likely cooperates in a synergistic manner with oncogenic *EGFR* (Figure 5D). These differences in tumorigenesis resulted in a significantly impaired survival in the *C/EGFR^DEL^/Dok2* ^*-/-*^ animals compared to *C*/EGFR^DEL^/Dok2^+/+^ mice (Figure 5E; P < 0.05, n = 29 *Dok2* ^*+/+*^ and n = 39 *Dok2* ^*-/-*^). 

**Figure 6:**
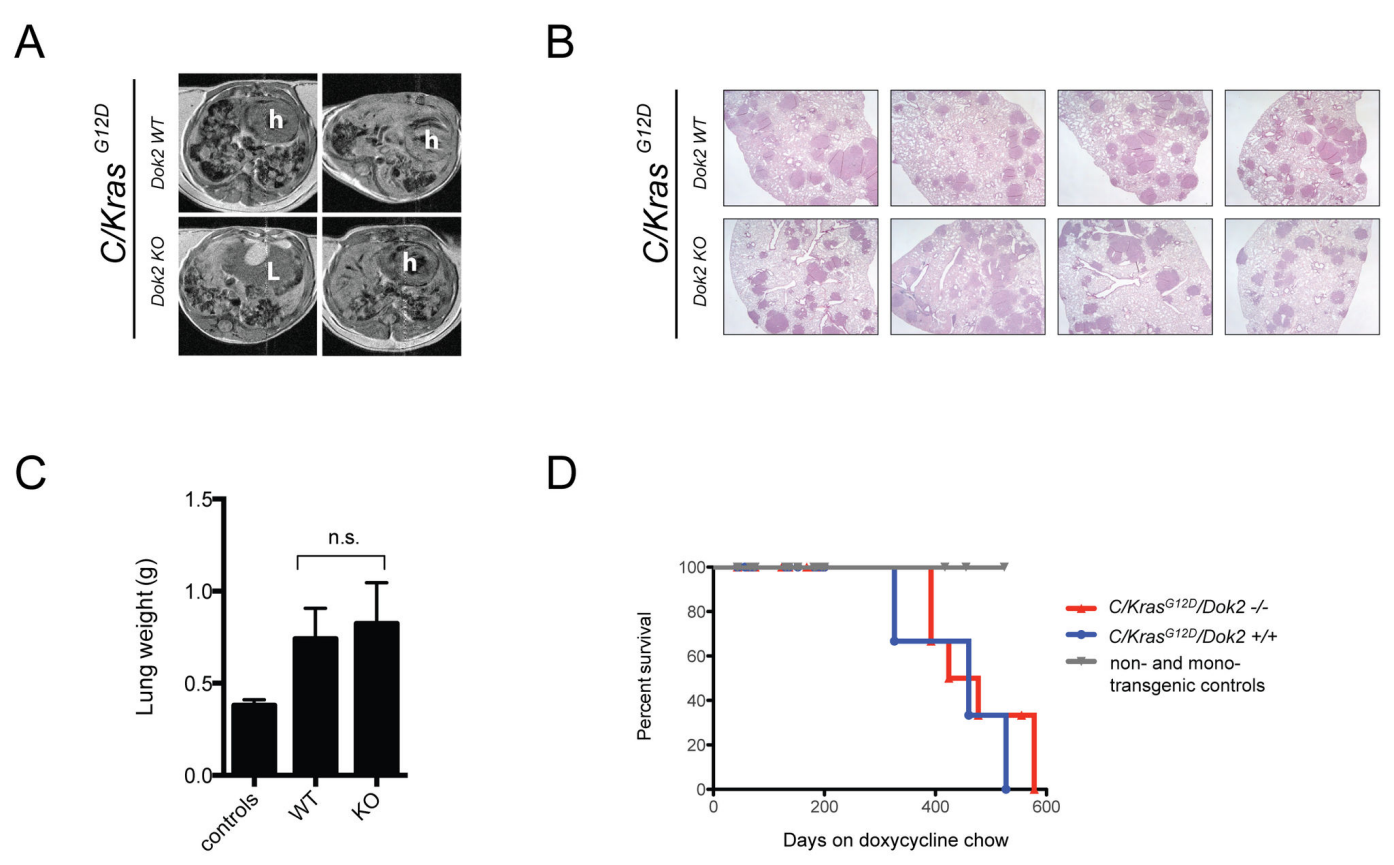
Loss of *Dok2* fails to impact *Kras*-mutant lung tumorigenesis. (**A**) MR images of the lungs of *C/Kras^G12D^/Dok2* ^*+/+*^ and *C/Kras^G12D^/Dok2* ^-/-^ lungs after 5 months of doxycycline induction. h, heart. L, liver. (**B**) H&E staining of lungs from *C/Kras^G12D^/Dok2* ^*+/+*^ and *C/Kras^G12D^/Dok2* ^-/-^ after 5 months of doxycycline treatment. 20X total magnification. For each genotype, four lung lobes from a single representative mouse are shown. (**C**) Lung weight data from 4-5 month old animals. Mean + SEM is shown from n = 6 control (non-transgenic) mice and n = 4 *C/Kras^G12D^/Dok2* ^*+/+*^ and *C/Kras^G12D^/Dok2* ^-/-^ animals. (**D**) Kaplan-Meier curve showing survival data from *C/Kras^G12D^/Dok2* ^*+/+*^ and *C/Kras^G12D^/Dok2* ^-/-^ and non- or mono-transgenic controls (*Dok2* +/+ and -/-) treated with doxycycline for the indicated times.

Next we tested whether loss of *Dok2* enhances lung tumorigenesis in a mouse model of *Kras-*driven tumorigenesis. *C/Kras* ^*G12D*^ bitransgenic mice express the oncogenic *Kras* mutation, *Kras* ^*G12D*^, specifically in the lung and rapidly develop hundreds of small adenomas and adenocarcinoma tumors after doxycycline administration [23]. Both *C/Kras^G12D^/Dok2* ^*+/+*^and *C/KRAS^G12D^/Dok2* ^*-/-*^ mice developed numerous tumors. At very early time points less than 3 months post-doxycycline administration we did observe an increase in tumor number in *Dok2* KO mice (data not shown). However, at 4-5 months of doxycycline induction we did not detect a difference in tumor burden by MRI or lung weight between the two groups (Figure 6A-C). Pathological review revealed no differences between the histology of the two cohorts. Both *Dok2* wild-type and KO *C/Kras* ^*G12D*^ mice developed typical grade II adenomas with uniform nuclei. A few tumors in each genotype exhibited nuclear pleomorphism. Furthermore, there was no difference in overall survival between the two cohorts (Figure 6D). We therefore conclude that it is unlikely that loss of *Dok2* provides a marked selective advantage for *Kras*-mutant lung cells.

## Discussion

Several lines of evidence support a role for *DOK2* in suppression of lung tumorigenesis driven by oncogenic *EGFR*. First, we observe loss of *DOK2* in human lung adenocarcinoma and association of this feature with somatic mutation of *EGFR*. Second, *DOK2* overexpression inhibited the tumor-forming ability of lung adenocarcinoma cells harboring an *EGFR* mutation. Third, we demonstrate in mouse models that loss of *Dok2* promotes lung tumorigenesis initiated by oncogenic *EGFR*. Fourth, DOK2 is constitutively membrane-associated and bound to EGFR in cells expressing mutant, oncogenic *EGFR*. Fifth, DOK2 can physically interact with EGFR and suppress its downstream signaling [12,17], an observation that we confirm for the first time in the context of *EGFR* mutation. 

**Figure 7:**
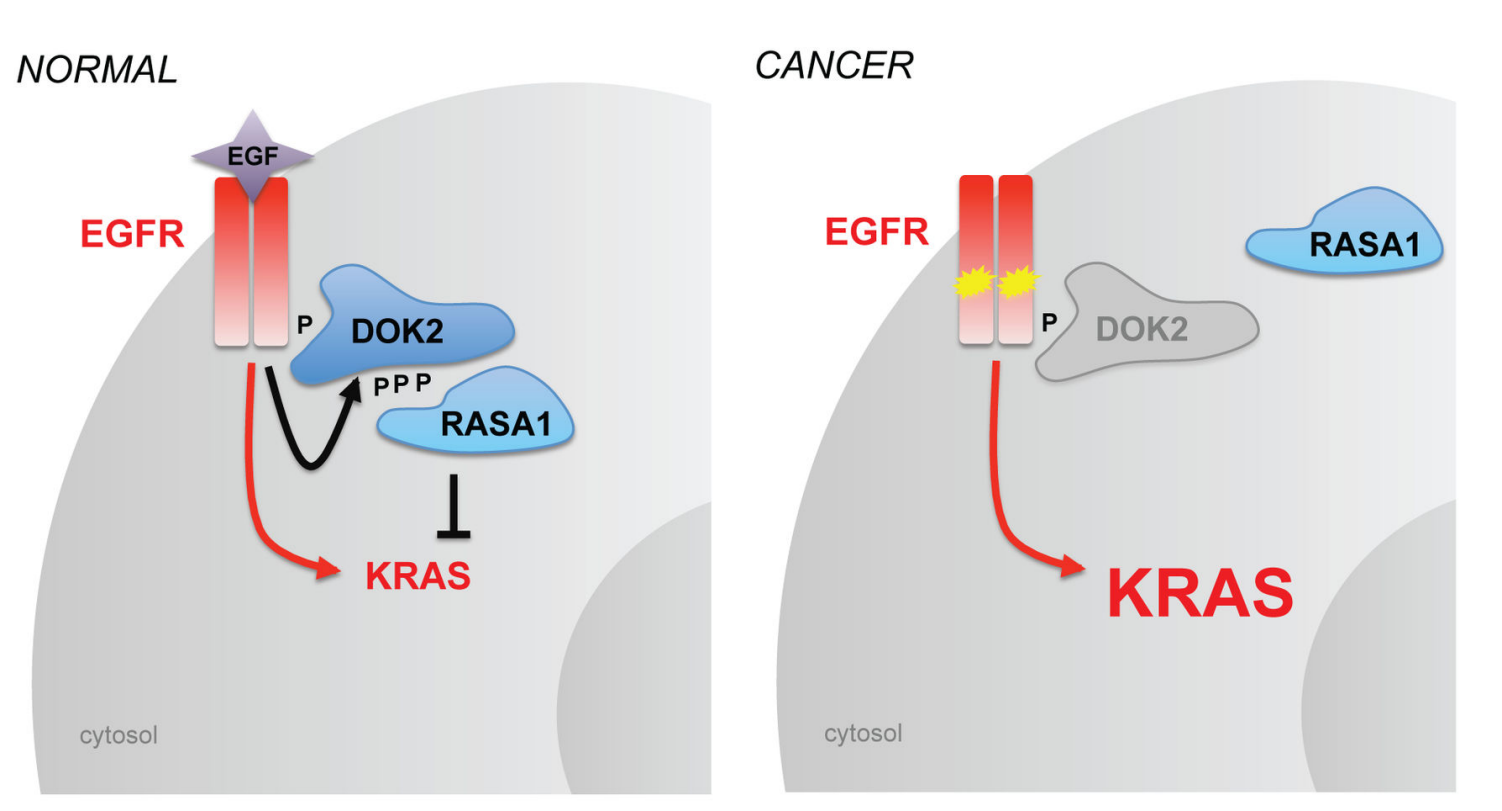
Model of DOK2 function in normal lung and lung adenocarcinoma. Left, physiological function of DOK2 in normal lung cells in regulation of the EGFR pathway. EGFR activation via ligand binding of EGF induces both KRAS activation (red arrow) and a negative feedback loop involving recruitment of DOK2 (black arrow) and RASA1. Right, pathogenic loss of DOK2 and deregulation of the EGFR signaling pathway. Loss of DOK2 (gray) results in decreased recruitment of RASA1 (blue), allowing enhanced downstream activation of KRAS following EGFR mutation and activation (yellow).

One possible mechanism of DOK2 function involves DOK-mediated recruitment of RASA1, which in turn facilitates RASA1-induced inactivation of RAS. The ability of DOK2 to inhibit EGF-induced activity of wild-type RAS and the fact that DOK2 is constitutively bound to RASA1 in cells harboring EGFR^L858R^ suggest that at least part of DOK2’s tumor suppressive function is to suppress RAS activation via RASA1. In contrast to the effect of loss of *DOK2* in tumors with an *EGFR* mutation, loss of *DOK2* is not consistently associated with *KRAS* mutation in human lung adenocarcinoma and we do not observe an enhancement of tumor formation in *Kras*-mutant mice lacking *Dok2*. Thus the tumor suppressive function of DOK2 in the context of EGFR-RAS signaling lies upstream of RAS, likely through DOK2’s canonical effector, RASA1. Together our data support a model (Figure 7) in which loss of *DOK2* impairs negative feedback on oncogenic signaling, leading to enhanced EGFR-RAS signaling and cancer. It remains possible that, in some contexts, RASA1-independent functions of DOK2 may allow DOK2-mediated regulation in parallel or downstream of activated RAS. Similarly, continued inhibition of the remaining wild-type RAS in a *RAS*-mutant cell may provide a biologically relevant level of signal inhibition in some contexts. We previously observed suppression of growth of an *NRAS*-mutant cell line by DOK2 [19] and so the relationship of DOK2 to mutated *RAS* genes requires further investigation.

The findings presented here clearly indicate that loss of *DOK2* enhances *EGFR*-driven lung tumorigenesis. However, *EGFR* mutation can nonetheless induce tumor formation in the presence of an apparently intact *DOK2* gene. We observe human lung adenocarcinomas without copy number loss of *DOK2* (Figure 1), and expression of mutated *EGFR* induces tumor formation in *Dok2* wild-type mice, albeit more slowly and less effectively than in a *Dok2* null genetic context (Figure 5). Additional analyses will be required to determine whether *DOK2* is indeed fully functional in those settings or whether the gene may have been inactivated by other mechanisms, such as methylation or mutation. It is also possible that EGFR is able to override DOK2-mediated inhibition via mechanisms such as increased expression of EGFR driven by amplification of the mutant *EGFR* allele. Indeed, amplification of mutated *EGFR* is frequently observed in tumors [24]. Another possibility is that DOK2-mediated negative feedback may be disrupted via alteration of other members of the pathway. For example, SHC1 is an SH2-domain containing protein that recruits RASGEFs to RAS and binds to the same pTyr sequences on EGFR as DOK2. Overexpression of SHC1 could conceivably override the ability of DOK2 to be recruited to activated EGFR. Although some doubt has been cast on a competitive-binding model of DOK2/SHC1 regulation of RAS [17], additional investigation is required to definitively rule out this model. Alternatively, loss or mutation of DOK2’s effector, *RASA1*, would be predicted to disrupt DOK2’s ability to suppress EGFR signaling. Recent sequencing data shows that *RASA1* mutations are observed in lung adenocarcinoma (COSMIC database), so further effort should be directed towards understanding the relationship between RASA1, DOK2, EGFR, and KRAS in lung adenocarcinoma. 

On the other hand, loss of *DOK2* is also observed in *EGFR* wild-type tumors, and *Dok2* null mice do spontaneously develop lung adenocarcinoma [17]. It is therefore possible such tumors have alterations in other receptor tyrosine kinases that may also be regulated by DOK2 via binding of DOK2’s PTB domain to phosphotyrosines on those receptors. Many other RTKs are known to play a role in lung adenocarcinoma, including ALK and ERBB2, and it is interesting to speculate that DOK2 may also inhibit lung tumorigenesis driven by those oncogenes. It is also possible that loss of *DOK2* on its own or in combination with loss of other tumor suppressors (e.g. *DUSP4* which is located with *DOK2* in the same frequently deleted locus at 8p21.3 [24]) is able to trigger a robust activation of the MAPK pathway hence favoring lung tumorigenesis.

Another unanswered question is the clinical consequence of *DOK2* genomic loss on patient outcome. Current sample numbers limit the ability to definitively describe the effect of loss of *DOK2* on survival, and no data exist on the relationship of *DOK2* to acquired resistance to EGFR inhibition. The continued characterization of patient samples with integrated genomic, expression, and protein data will no doubt provide further insight into the regulation of *EGFR*-mutant lung adenocarcinoma.

## Materials and Methods

### Ethics statement

All animal studies were reviewed and approved by the Institutional Animal Care and Use Committees at Memorial Sloan-Kettering Cancer Center and Beth Israel Deaconess Medical Center. Animals were euthanized at signs of distress or poor body condition to ameliorate pain and distress associated with tumor formation.

### Human tumor analyses

Patient information, array CGH analysis, and mutation identification in 199 primary human adenocarcinoma samples are as previously described [19,20]. For the TCGA analysis, data from 230 lung adenocarcinomas was downloaded (http://tcga-data.nci.nih.sgov/‎) or analyzed using the MSKCC cbio portal (http://cbioportal.org). GISTIC [25] was used to define heterozygous losses.

### Cell lines

NIH3T3, HEK293, NCI-H1975, A549, and ecotropic Phoenix cells were purchased from the American Type Culture Collection (ATCC). 

### Co-immunoprecipitation and Western blotting

HEK293 cells were co-transfected with pCMV-DOK2 and pcDNA3.1-EGFR^WT^, pcDNA3.1-EGFR^L858R^ or pcDNA-HA-HRAS^G12V^, then incubated for 24 hours. Cells were serum starved overnight in medium containing 0.1% FBS, stimulated with 50 ng/ml EGF for 2-5 minutes, then lysed in a co-immunoprecipitation buffer (150 mM NaCl; 1mM EDTA; 50 mM HEPES, pH 7.5; 1% Triton X-100; 10% glycerol; 1mM beta-glycerol phosphate; 1mM Na_3_VO_4_; 1mM NaF; and Roche “Complete” protease inhibitor). Antibodies used for the co-immunoprecipitation and Western blot were α-DOK2 (E10, Santa Cruz), α-EGFR (13, BD Biosciences), α-phosphotyrosine (PY99, Santa Cruz Biotechnology), anti-pan RAS (Ab-3, Calbiochem), anti-RasGAP (Santa Cruz Biotechnology, sc-63), and anti-tubulin (Sigma). Antibodies used for Western blot (Figure 2B) were α-EGFR^L858R^ (a kind gift of Dr. William Pao, Vanderbilt University), α-EGFR (BD), and anti-β-actin (Sigma). 

### Generation of NIH3T3 cell lines stably overexpressing EGFR

Constructs pBabe-puro-EGFR and pBabe-puro-EGFR^L858R^, a kind gift of Jeonghee Cho and Matthew Meyerson (DFCI), were used to transfect ecotropic Phoenix cells. 48 and 72 hours post-transfection, culture supernatants were filtered, supplemented with polybrene, and added to NIH3T3 cells. 24 hours after the last infection, cells were selected for 2 days in media containing 2 μg/ml puromycin before use in experiments. 

### Immunofluorescence

NIH3T3 cells were cultured on chamber slides, serum starved overnight in medium containing 0.1% FBS, then either left unstimulated or stimulated with EGF (50 ng/ml) for 5 minutes. Cells were fixed with 4% paraformaldehyde (PFA) and permeabilized with PBS containing 0.1% Triton X-100. Cells were incubated with primary antibodies overnight at 4°C before secondary detection using Alexafluor-conjugated secondary antibodies (Invitrogen) and mounting and nuclei counterstaining using Vectashield with DAPI (Vector Labs). Primary antibodies used were: anti-EGFR (BD), anti-DOK2 (E10, Santa Cruz), or anti-FLAG. 

### Fluorescent Microscopy

Images were acquired with either with a Nikon Eclipse TE300 microscope (Figure 2B) or a Zeiss LSM 510 Meta Confocal Microscope (Figure 2C). Post-acquisition image processing was performed using Adobe Photoshop. 

### Murine models

CCSP-rtTA; tetO-EGFR^DEL^ (*C/EGFR* ^*DEL*^) and CCSP-rtTA; tetO-Kras^G12D^ (*C/Kras* ^*G12D*^) bitransgenic mice were crossed into the *Dok2* KO strain. All mouse strains have been previously described [14,22,23]. Mice heterozygous for both transgenes were used for all experiments, and monotransgenic or nontransgenic mice were used as negative controls. Cohorts of mice were continuously kept on a diet containing doxycycline (625 ppm; Harlan-Teklad) from weaning (3 weeks) until the experimental endpoint. 

### Magnetic resonance imaging

MR imaging experiments were performed at 4.7 Tesla (BioSpec 47/40; Bruker Biospin) using a transmit/receive birdcage coil (inner diameter, 30 mm). Animals were anesthetized with 1-2% isofluorane (Florane; Baxter) via a nose cone, and placed in prone position, headfirst, with the thorax centered with respect to the center of the coil. Cardiac and respiratory gating was used to trigger multislice acquisition of a single line of k space in each cardiac cycle, during exhalation interval of respiration. Axial images of lungs were acquired using a spin echo pulse sequence with repetition time (TR) = 1000 ms, echo time (TE) = 12 ms, matrix size = 256 x 256, field of view (FOV) = 2.56 x 2.56 cm, and slice thickness = 1 mm.

### Histology and Immunohistochemistry

Animals were anesthetized with Avertin and then perfused with 10 ml cold PBS through the left ventricle of the heart. Lungs were inflated via intratracheal injection of 4% PFA, dissected, and fixed in 4% PFA overnight before subsequent processing, paraffin embedding, and H&E staining (Histoserv, Inc or BIDMC Histology Core). Immunohistochemistry was performed using a Ki67 antibody (Vector labs VP-451). Histopathology was reviewed by R.T.B. 

### RAS activity assay

RAS activation was measured using GST-RBD (RBD, RAS-binding domain of RAF) pull-down assays as previously described [14]. Briefly, cells (either unstimulated or stimulated with 50 ng/ml EGF) were lysed in lysis buffer (50 mM Tris-HCl, pH=7.4; 150 mM NaCl; 1% Triton X-100; 10% glycerol; 0.25% sodium deoxycholate; 10mM MgCl_2_; 1mM EDTA). Equal amounts of cell lysates were incubated with bacterially expressed GST-RBD coupled to glutathione sepharose beads (Amersham). Beads were washed with lysis buffer, and GST-RBD associated RAS (RAS-GTP) and total RAS in cell lysates were detected by Western blotting using a pan-RAS antibody (BD Transduction Laboratories). Relative RAS activity was quantified by normalizing the amount of RAS-GTP to the total amount of RAS in cell lysates and then normalizing to the value of 1.0 for control cells.

### Cell culture and EGF treatment

A549 and NCI-H1975 cells were purchased from American Type Culture Collection and cultured in RPMI 1640 supplemented with 10% fetal bovine serum (FBS) at 37 °C/5% CO_2_. For the EGF treatment, cells were serum starved in RPMI plus 0.1% FBS for 24h, and then stimulated with 100ng/ml EGF (Invitrogen) for the indicated time. At the end of the stimulation, cells were immediately washed with ice-cold PBS and lysed in RIPA buffer (Sigma) containing complete EDTA-free protease and phosphatase inhibitor cocktails (Roche). Cell debris was pelleted, and supernatants containing the whole cell lysates were analyzed on SDS-polyacrylamide gels. 

### *In vivo* tumorigenesis assay.

6-week-old male athymic nude mice (NCr-nu/nu) were purchased from Taconic and inoculated subcutaneously in the right flank with 5 × 10^6^ A549 or 4 × 10^6^ NCI-H1975 cells in 50ul 100% Matrigel (BD Biosciences). Tumor size was measured weekly using a caliper, and tumor volume was determined using the standard formula *L* X W^2^ X 0.5, where L is the longest diameter and W is the shortest diameter, as previously described [19].

### Statistics

For analysis of quantitative data, datasets were compared using unpaired two-tailed Student’s t-tests with a *P* value less than 0.05 considered significant. Datasets with unequal variances were analyzed as above but with application of Welch’s correction. For analysis of categorical data, 2x2 contingency tables were constructed and datasets were compared using a Fisher’s exact test with a *P* value less than 0.05 considered significant. For survival data, curves were compared using a Log-Rank Mantel-Cox test. All statistical tests were executed using GraphPad Prism software.

## Supporting Information

Figure S1 **DOK2 inhibits EGF-induced RAS and ERK activation.** (**A**) RAS activity assay measuring EGF-induced activation of RAS in HEK293T cells transfected with empty vector control or FLAG-*DOK2*. An anti-panRAS antibody was used to detect RBD-bound active RAS (top panel) or total RAS in lysates (second panel). Numbers below the two panels represent relative RAS activity quantified by normalizing the amount of RAS-GTP to the total amount of RAS in cell lysates, and then to the value of 1 for control cells. Lower panels, Western blot analysis of total lysates using anti-FLAG (DOK2) or anti-ERK2 (loading control) antibodies. (**B**) Western blot of lysates from (A) using anti-phospho-ERK (top panel) or anti-total ERK2 (loading control) antibodies. Data shown is representative from at least three independent experiments.
(TIF)
