# Supplementary figures and images for: DOK2 Inhibits EGFR-Mutated Lung Adenocarcinoma
Source: PLoS One. 2013 Nov 8;8(11):e79526. doi: 10.1371/journal.pone.0079526 (PMC3821857; doi:10.1371/journal.pone.0079526)

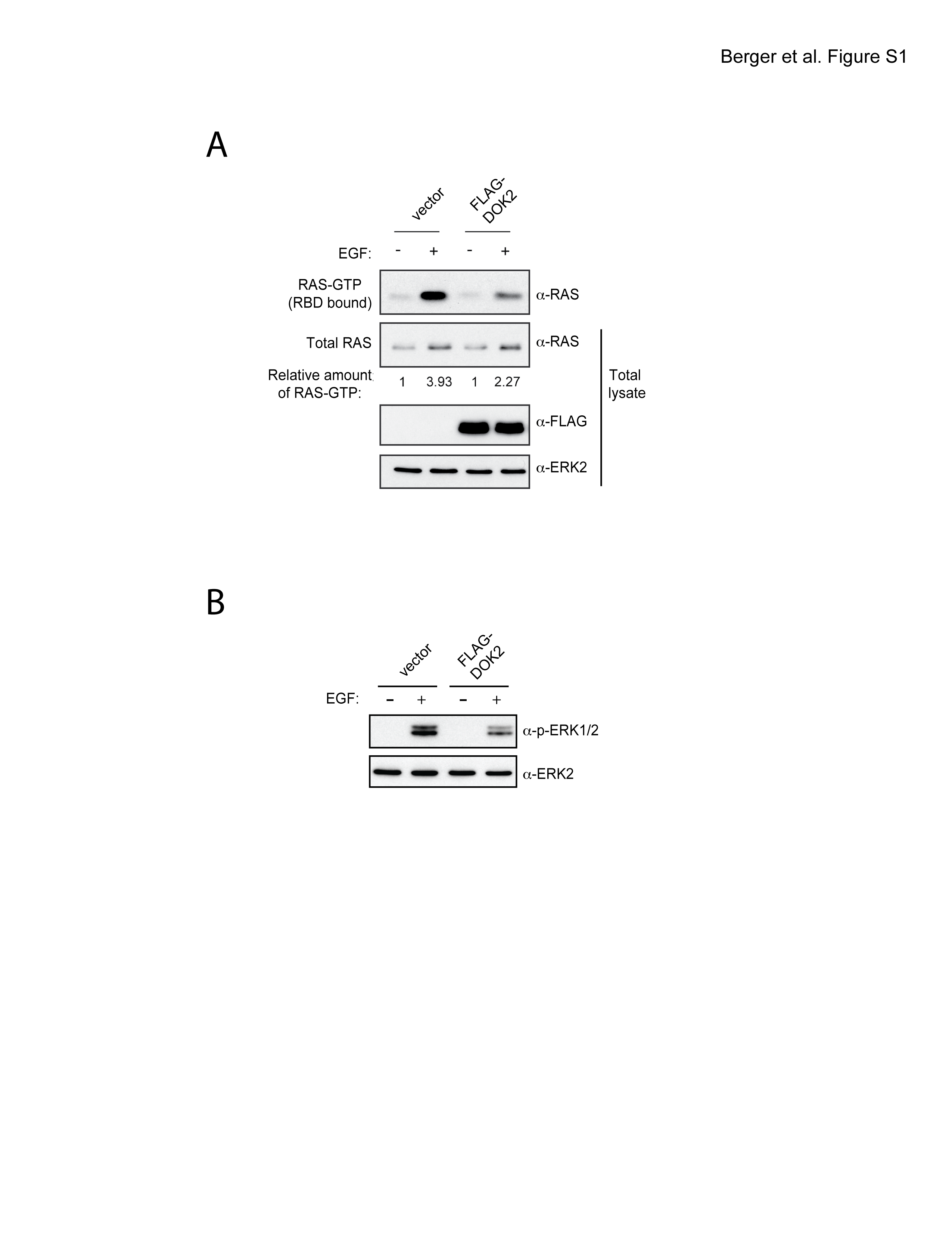

Supplement: Figure S1 — DOK2 inhibits EGF-induced RAS and ERK activation. (A) RAS activity assay measuring EGF-induced activation of RAS in HEK293T cells transfected with empty vector control or FLAG-DOK2. An anti-panRAS antibody was used to detect RBD-bound active RAS (top panel) or total RAS in lysates (second panel). Numbers below the two panels represent relative RAS activity quantified by normalizing the amount of RAS-GTP to the total amount of RAS in cell lysates, and then to the value of 1 for control cells. Lower panels, Western blot analysis of total lysates using anti-FLAG (DOK2) or anti-ERK2 (loading control) antibodies. (B) Western blot of lysates from (A) using anti-phospho-ERK (top panel) or anti-total ERK2 (loading control) antibodies. Data shown is representative from at least three independent experiments. (TIF) [file pone.0079526.s001.tif]
